# Return to work of breast cancer survivors: a systematic review of intervention studies

**DOI:** 10.1186/1471-2407-9-117

**Published:** 2009-04-21

**Authors:** JL Hoving, MLA Broekhuizen, MHW Frings-Dresen

**Affiliations:** 1 Coronel Institute of Occupational Health and Research Center for Insurance Medicine, Academic Medical Center, University of Amsterdam, Amsterdam, the Netherlands

## Abstract

**Background:**

Breast cancer management has improved dramatically in the past three decades and as a result, a population of working age women is breast cancer survivor. Interventions for breast cancer survivors have shown improvements in quality of life and in physical and psychological states. In contrast, efforts aimed at stimulating re-employment and return-to-work interventions for breast cancer survivors have not kept pace. The objective of this review was to study the effects and characteristics of intervention studies on breast cancer survivors in which the outcome was return to work.

**Methods:**

The Cochrane Controlled Trials Register (The Cochrane Library, Issue 4, 2006), Medline, Ovid, EMBASE and PsychInfo were systematically searched for studies conducted between 1970 to February 2007. Intervention studies for female breast cancer survivors that were focused on return to work were included.

**Results:**

Our search strategy identified 5219 studies. Four studies out of 100 potentially relevant abstracts were selected and included 46–317 employed women who had had mastectomy, adjuvant therapy and rehabilitation, with the outcome return to work. The intervention programs focused on improvement of physical, psychological and social recovery. Although a substantial percentage (between 75% to 85%) of patients included in these studies returned to work after rehabilitation, it is not clear whether this proportion would have been lower for patients without counseling or exercise, or any other interventions, as three out of four studies did not include a comparison group.

**Conclusion:**

The most important finding of this review is the lack of methodologically sound intervention studies on breast cancer survivors with the outcome return to work. Using evidence from qualitative and observational studies on cancer and the good results of intervention studies on return to work programs and vocational rehabilitation, return to work interventions for breast cancer survivors should be further developed and evaluated.

## Background

Breast cancer detection and care management have undergone dramatic changes in the past three decades. Survival of breast cancer has become more frequent because women are increasingly diagnosed with early-stage disease and screening. Treatment is focused on curing the disease and preventing relapse due to metastatic disease. The overall five-year survival rate is now above 85% in The Netherlands [1] and USA [2].

These major advances in detecting and managing breast cancer have led to the treatment of women who are more likely to be of working age. For some women, breast cancer may impose an economic hardship because it causes them to leave their jobs [3]. Compared with women of similar age without a history of cancer, a slightly higher proportion of cancer survivors experience reduced work ability, temporarily as well as permanently, due to retirement or disability [4-6]. Being unable to return to work after cancer treatment, frequent or prolonged work absenteeism, or problems with work performance may have a substantial economic impact on the survivor and her family [7]. Job absenteeism is an important economic outcome because days missed from work are costly to both the employer and the employee. Moreover, it is stated that the longer people are absent from their jobs, the lower the likelihood is that they will ever return to work [8]. Return to work after breast cancer is important, not only from a societal point of view, but also for the rehabilitation of the cancer survivor [9,10]. Moreover, returning to or maintaining employment after cancer is important for survivors' quality of life, including physical and mental health [11].

In recent years, rehabilitation therapies have been developed for breast cancer survivors that focus on quality of life including physical and mental health. For example, interventions reported in observational studies that aim to improve quality of life for breast cancer survivors range from inpatient (spa) rehabilitation to telephone counselling [12-16]. The role of exercise for breast cancer patients has been examined for breast cancer-related side effects which may be especially important in patients who receive adjuvant treatment [17-19], though the number of studies is low and different types of exercise are used.

Most of these interventions show some improvement on quality of life or on other physical and psychological outcomes, such as cancer related fatigue, but do not pay attention to the aspect of work which is considered to be an important contributor to quality of life. For this reason we conducted a systematic review with the objective to study the effects and characteristics of intervention studies on breast cancer survivors in which the outcome was return to work.

## Methods

### Identification of studies

The literature search included a systematic review of the following electronic bibliographic databases: Medline – Ovid, EMBASE, PsychInfo and the Cochrane Controlled Trials Register (The Cochrane Library, Issue 4, 2006). These databases were studied from 1970 to February 2007. Reference lists of relevant articles were scanned for any other relevant studies.

### Selection of studies

All searches were conducted by one researcher (MLAB) and checked by another (JLH). All abstracts and titles were initially screened for relevance, i.e. whether it concerned an intervention study of female breast cancer survivors, and measured return to work.

The details of the search are listed in Appendix A.

The following inclusion criteria were applied for full text articles.

#### (i) Types of Studies

Randomised controlled studies (RCT), cohort studies and observational studies.

#### (ii) Types of Participants

Women who were diagnosed and had survived breast cancer with or without adjuvant therapy (i.e., chemotherapy, radiotherapy) during the intervention period.

#### (iii)Types if Interventions

All non-drug interventions.

#### (iv) Types of Outcome Measures

Studies that measured work-related outcomes such as: (a) return to work, (b) absenteeism, (c) work disability, (d) sick leave or (e) employment status.

### Data Extraction

Data were extracted from the included papers by one reviewer (MLAB) and checked for accuracy by the second reviewer (JLH). Disagreement in data extraction between reviewers was solved by consensus. The extracted data included; first author, year and place of study, design, participant characteristics, description of intervention(s), length of follow up, outcomes and the effect of the interventions on return to work.

## Results

Our search strategy revealed 5219 studies. Among these studies, 100 potentially relevant abstracts were identified. Following review of the full text articles, four full-length articles remained, each of which was eligible for inclusion [20-23]. The remaining 96 articles were excluded, mainly because these studies did not include work-related outcomes.

### Study characteristics

Table 1 summarises the characteristics of the included studies [20-23]. In these four studies, 46–317 women who were working before the diagnosis of breast cancer were evaluated after a programme consisting of physical exercise and psychological counselling given individually or in group sessions by a specialized nurse or a team of specialists. Three out of four studies were published before 1990, and only one used a control group. Two studies described the same intervention, follow-up and outcomes, but in different hospital settings. The aim of all four studies was to improve physical and social recovery and adaptation to breast loss. Work status was reported as return to work percentages in all studies.

**Table 1:** Summary characteristics of included studies

Study	Participants	Number of subjects enrolled	Description intervention (s)	Length of follow up and outcomes	Proportion returning to work
Maguire, 1983, UK Controlled trial	Women undergoing modified radical mastectomy and full axillary clearance Setting: surgical unit Age: not mentioned	N = 152 women, N = 75 in counselling group and N = 77 in care as usual group Work status before cancer: Counselling group N = 42 Care as usual group N = 46	Counselling group: Counselling content: - inpatient care: advice on exercises, encouragement to look at scar and discuss feelings, discuss possible external breast prosthesis - at follow-up: encouragement to return to work and become socially active Counselling frequency: - inpatient care: pre- and post- mastectomy - at follow-up: after discharge, every 2 months, home visits until it was clear patient had adapted well Counselling was given by a specialist nurse Care as usual group: Received usual care provided by surgical unit	Follow up: a few days after surgery, 3, 12 and 18 months. Outcomes: assessed using a semi-structured interview and physical assessment: - return to work - house work - social adjustment - persisting problems arm: pain, swelling, disability and range of motion - response to scar, prosthesis and breast loss	Return to work: - Counselling group: 76% (32 out of 42 patients) between 12–18 months - Care as usual group: 54% (25 out of 46 patients) between 12–18 months For return to work versus not χ^2 ^= 4.59, p < 0.05

Fismen, 2000, Norway Non-Controlled study	Women who had received surgical treatment, chemotherapy and radiation therapy for breast cancer (stage 1 and 2), in combination with rehabilitation Setting: surgical unit Age: 31–66 (mean 49 years)	N = 50 women Work status before cancer: N = 46	Content: Training of physical capacity; cognitive group discussions in small groups (2–10 persons) Examinations of psychological and physical status were performed before rehabilitation, after rehabilitation and after follow-up Frequency: 3 weeks rehabilitation program, 3 months at home and 1 week follow up at rehabilitation centre.	Follow-up: - after 3 weeks rehabilitation - after 3 months at home - after 1 week follow-up in rehabilitation centre Outcomes: assessed by questionnaires: - return to work - mental state (depression, anxiety, mood states) - quality of life and pain. In addition, assessment of lymph edema and VO2 max	Return to work: 78% (36 out of 46 patients) during the 3 month follow-up

Sachs, 1980, USA Non-Controlled study	Women who underwent modified radical mastectomy and full axillary clearance (Mt. Sinai data) and rehabilitation Setting: surgical unit Age: not mentioned	N = 107 women Work status before cancer: not mentioned	Content: - counselling: emotional support, encouragement to look at scar, discuss feelings, discuss possible external breast prosthesis, encouragement to return to work and become socially active - exercises Frequency: 90 minutes a day, 5 days a week until discharge from hospital - counselling: on alternating days - exercises: daily Professionals involved: A team consisting of a physical therapist, a nurse, a social worker, and a Reach-To-Recovery volunteer for 3 days each week Counselling: - mastectomy rehabilitation nurse: visits with patient before and after operation, informs patient and family of programme, lends moral support - social worker: leads groups sessions in the programme Exercises: - demonstrated by physical therapist and performed daily The physician was a member of the team	Follow up: 90 days after discharge a questionnaire was sent to the participating patients Outcomes: assessed using questionnaires: - return to work - resumption of normal activities - physical recovery and rehabilitation - emotional stress	Return to work: 85% of patients. Average duration out of work: 5.9 weeks

Winick, 1977, USA Non-Controlled study	Women who underwent *Simple or total mastectomy: **Modified radical mastectomy: + axillary lymph nodes and part pectoral muscle ***Standard or radical mastectomy: + lymph nodes and underlying pectoral muscles ****Extended radical mastectomy; + all axillary lymph nodes, underlying pectoral muscles, and medial segment chest wall All women received rehabilitation Setting: surgical unit Age: 20–91 (mean 56 years)	N = 863 women Work status before cancer: N = 317 employed full time	Same Post Mastectomy Rehabilitation Programme as study by Sachs et al.	Follow up: 3 months after discharge Outcomes: assessed using a questionnaire sent to patients: - return to work - resumption of normal activities - physical recovery and rehabilitation - emotional stress and personal relationship adjustment difficulties	Return to work: 75% of patients (237 out of 317 patients) full time return to work within 3 months: *: (n = 2), 100%, average weeks to resume: 3 **: (n = 50), 78%, average weeks to resume: 7.1 ***: (n = 241), 75%, average weeks to resume: 8.6 ****: (n = 24), 63%, average weeks to resume: 9.5

The only controlled study was reported by Maguire et al (1983)[20] in patients who had radical mastectomy and full axillary clearance. Half the weeks during a 24-month period were designated as 'counselling' weeks and the other half as 'control' weeks using a random number table. Women admitted for mastectomy in these weeks were assigned to the selected group for the duration of the study.

The counselling group comprised 75 women of which 42 worked before being diagnosed with breast cancer. These patients received individual care given by a specialist nurse who gave them help before and after surgery, in addition to the usual care of the surgical unit. During hospitalisation, counselling and advice was provided by showing patients how to carry out exercises designed to restore full mobility to the arm, and by encouraging the patient to look at the scar and discuss feelings regarding losing the breast. After discharge (length hospital stay was not specified), the intervention was continued at the woman's home. The specialized nurse checked the exercise program clarified how the woman felt about the scar and breast loss, and tried to ensure that the woman obtained a satisfactory prosthesis. The specialist nurse emphasized the value of the woman being open with her partner about the diagnosis and surgery. Furthermore, return to work and engagement in social activities were encouraged. The nurse followed each woman up every 2 months to monitor her progress unless it was already clear that the patient had adapted well. Details on the number of the visits were not specified.

The control group comprised 77 women, of whom 46 worked before the diagnosis of breast cancer. They received only the care normally given by the surgical unit. No specification of the usual care was given by Maguire et al [20]. The results of this study showed more favourable return-to-work rates in the counselling group (n = 32, 76%) compared with the control group (n = 25, 54%) within the 12 to 18 month follow-up period (p < 0.05).

Other outcomes, including response to scar, prosthesis and breast loss, showed that significantly more counselling than control patients displayed better results and had adapted well. With regard to housework and social adjustment, control subjects were less active than the counselled group.

The other three studies were non-controlled intervention studies [21-23]. Two studies referred to the same intervention, the "The Post Mastectomy Rehabilitation Group Program" (PMRG), in different hospital settings and different patients [22,23]. Both studies used a before-after design and reported that PMRG stimulated women to return to work and to resume normal activities [22,23].

Sachs et al (1980) included 107 patients who underwent only one type of mastectomy – a modified radical mastectomy with full axillary clearance (data Mt. Sinai Hospital) [22]. Pre-cancer work status was not mentioned in the study by Sachs [22]. Winick and Robbins (1977) included patients with different types of mastectomies, ranging from simple mastectomy, modified radical mastectomy, radical mastectomy and extended radical mastectomy [23]. In their study, 863 women were included of whom 317 women worked before the diagnosis of breast cancer.

The basic PMRG program included 'all' in-patient breast cancer patients that met for 90 minutes a day, five days a week. The multidisciplinary approach comprised a series of structural exercises, information and group therapy sessions which were conducted by a social worker, nurse or physical therapist. Additionally, one-on-one counselling by a volunteer from Reach to Recovery (not specified) was given. A key aspect of the PMRG was the manner in which the team of physicians, nurses, social workers and physical therapists operated. Each professional knew what the other team members did and needed to do. Moreover, the rest of the team understood how best to involve the physician in making the program work.

The women in these two studies were encouraged to attend the program on their second or third post-operative day, daily, until they were discharged [22,23]. Exercises were demonstrated by the physical therapist and lasted about half the time of most sessions. These exercises usually began on the patient's second or third postoperative day. Most patients were involved in at least two sessions led by the nurse, who provided a description of the various types of mastectomy surgeries and reviewed the importance of specific points about hand and arm care. Self-care was particularly stressed and taught. The social worker led discussions on alternating days. It offered women in the group an opportunity to deal with their grief and fear in a setting with others experiencing similar emotions. In cases in which the woman's fear appeared more pervasive, individual counselling was given. During the counselling period, patients were encouraged to return to work and to become socially active again after discharge.

Winick and Robbins reported that the type of surgical procedure influenced recovery time, adaptation and return to work [23]. Patients who had less extensive surgery returned to work in less time compared to patients who had more extensive surgery. Two women had only a breast removed, a simple mastectomy and both returned to work in an average of 3 weeks. On the other hand, of the women who had extended radical mastectomy, which comprised of the removal of the breast with all axillary lymph nodes, the underlying pectoralis muscles and the medial segment of the chest overlying the internal mammary nodes and the internal mammary nodes, 63% (n = 24) returned to work in a average of 9,5 weeks.

The more recent, non-controlled study by Fismen et al (2000) reported an intervention study on 50 women who had undergone surgical treatment, chemotherapy and radiation therapy [21]. Sixty-four of these women worked before the diagnosis of breast cancer. The study describes a three-week rehabilitation program of physical and psychological interventions. It was followed by a three-month period at home and a one-week follow-up at the rehabilitation center. Training of physical capacity and cognitive group discussions were given in small groups (n = 2–10). After rehabilitation, 36 out of 46 (78%) women returned to work. In addition, there was an improvement in psychological parameters and an increase in the maximum oxygen uptake (from 67% to 77%) during the three-month follow-up.

## Discussion

The objective of this review was to study the effects and characteristics of intervention studies on breast cancer survivors in which the outcome was return to work. After an extensive literature review we identified only four intervention studies that measured return to work, three of which published more than 25 years ago. Although return-to-work rates around 75%–85% after up to 18 months follow up seem favourable, the lack of recently published high quality trials limits the strength of the evidence observed in these four studies.

It is not clear whether return to work would have been lower without rehabilitation consisting of counseling or exercise, or no intervention, as three out of four studies did not include a comparison group. A recent surveillance study by Bouknight et al (2006) in the US, found 82% of breast cancer survivors returned to work after 12 months [24]. These results are in contrast with a return to work rate of 56% reported in a Spanish breast cancer questionnaire study [25]. There is a wide range in disability outcomes among breast cancer survivors, even when they have had the same disease with equal severity [26]. Sick leave is on average about a year, but nevertheless varies from a couple of days to a couple of years. Though in the similar range, any comparisons between return to work rates observed in our review and recent survey studies are difficult to make as breast cancer survivor populations, medical procedures, rehabilitation strategies, and social security and health insurance provisions are not likely to be comparable.

On the other hand, return-to-work rates from more than two decades ago would likely be lower compared to today, as cancer related morbidity is lower and survival has significantly improved due to medical advances. Despite better medical treatment and physical and psychosocial rehabilitation, a five year follow up update of the Fismen et al study (2000) on the same study population reported that 23% of patients had died of cancer, depression and anxiety were similar to the situation directly post-rehabilitation, and almost half (44%) of the remaining patients had lost their job compared to 22% three months after surgery [27].

Of interest, the included studies [20,23] indicated that longer time needed to return to work was related to more extensive surgical procedures. In any case, time needed to return to work may be a more important outcome if absolute return-to-work rates continue to improve as some of these high return to work rates suggest.

Given the limitations of the three older studies and the somewhat sobering results of the recent Fisman study, we believe that return to work rates outcomes can be further improved. In addition to design issues and the quality of medical care, it is important to understand that return to work in this review was not the primary aim for any of the four studies included, but rather improvement of "physical and social recovery" in the context of a more 'traditional' rehabilitation context. In all four studies this included instructions or advice on specific exercises, for example to mobilise the shoulder or more general training of physical capacity, both of varying frequency and given in groups, individually or solely as a home assignment. In conjunction, counselling was part of a pre- and post operative care program and provided by a specialized nurse or social worker who provided support, encouragement, advice and openness to discuss feelings. Tailoring the exercise and counselling content specifically towards vocational outcomes (like return to work) was not apparent in any of the descriptions of these four studies and was, as explained earlier, not a specific long term goal.

Short et al (2005) showed that when work issues are addressed as a part of treatment, return to work is more successful after cancer [28]. Although the literature on breast cancer does not provide us with much information regarding interventions which are specifically targeted at vocational outcomes such as return to work, there is research from studies on cancer and other diseases in the occupational health literature which could be applied to improve return to work outcomes for breast cancer survivors. The issue of return to work should be addressed in intervention studies [29]. This includes more attention, information, support, and advice on work issues, not only by health care professionals but also by employers.

Rehabilitation needs of cancer patients focus around fatigue, reinforcing physical working capacity and psychosocial functioning [30]. In addition to prolonged physical or mental fatigue, qualitative research has also identified personal problems such as cognitive limitations, difficulty mobilising support, difficulty managing stress and anxiety, difficulty coping with a new self image, and changed attitude to work [24,31-34]. As mentioned above, both exercise and counselling seem good alternatives to deal with these problems that patients experience but could be further improved upon by including work resumption as a specific long term goal.

Work factors that could be positively targeted for interventions deal with, for example, good communication with co-workers and employers, early contact with the workplace, or work accommodations such as flexibility regarding work hours [34-36]. In addition, there is evidence that special arrangements made by the employer such as gradual return to work facilitates return to work [37]. Vocational rehabilitation or return-to-work programs target the barriers and facilitators for the return to work issues listed above and have been successfully applied in the area of rheumatoid arthritis [38] and other diseases [37,39].

Several rehabilitation strategies have proven valuable for patients with cancer or other diseases. For example, graded activity, a theory that promotes a step by step increase in activity, is beneficial for cancer patients to deal with fatigue and functioning [15]. In addition, workplace interventions such as work modifications and case management involving all stakeholders has been successfully applied for the return to work of workers with low back pain [40,41]. Also, cancer survivors may benefit from advice to structure the return to work process and use goal-setting during rehabilitation [42,43]. Verbeek and Spelten (2007) introduced a 10-step plan for return to work that was tested among cancer survivors at a radiotherapy department. The plan consists of a fixed scheme with the end-point return to work starting early in the rehabilitation process and in collaboration with the employer [29].

In this review the number of rehabilitation visits in the included studies is quite low. The duration of traditional rehabilitation is often limited to a maximum of about three months after surgery and this period may not be long enough to support patients who are in the process of returning to work. Therefore, if duration of functional capacity is to be really improved [19,44] it is suggested that rehabilitation programs are also of adequate duration, frequency and intensity. Additionally, changing psychosocial outcomes, behaviour in particular, may require more intensive intervention strategies during the complex return-to-work process over a longer period of time [43].

## Conclusion

In conclusion, the most important finding of this review is the lack of intervention studies that are focused on return to work. Using evidence from qualitative and observational studies on cancer and other diseases, and the good results of studies on return to work programs and vocational rehabilitation, return to work interventions for breast cancer survivors should be further developed and evaluated. Ultimately this may lead to a better quality of life and functioning, improve social economic outcomes, and a quicker return to work for breast cancer survivors.

## Competing interests

The authors declare that they have no competing interests. No financial support was sought in the production of this article.

## Authors' contributions

All authors have made substantial contributions to acquisition and analysis of data. JLH and MLAB have been involved in drafting the manuscript. MHW has been involved in the data extraction and in revising the manuscript critically for important intellectual content. Each author has participated sufficiently in the work and takes responsibility for appropriate portions of the content. All authors have read and have given final approval of the version to be published.

## Appendix A

A) Identification of breast cancer studies

1) (("breast neoplasms" [TIAB] NOT Medline [SB]) OR "breast neoplasms" [MeSH Terms] OR breast cancer [Text Word]) AND "humans" [MeSH Terms]

B) Identification of intervention studies

2) text words

effectiveness [tw] OR program [tw] OR intervention [tw] OR reduction [tw] OR effect$ [ti] OR evaluation [tw] OR decrease$ [tw] OR "prevention and control" [sh] OR measures [tw] OR improve$ [tiab] OR educat$ [tw] OR training [tw] OR rehabilitation [tw]

3) type design – use of PT and MESH

randomized controlled trial [pt] OR controlled clinical trial [pt] OR randomized controlled trials [mh] OR random allocation [mh] OR double-blind method [mh] OR single-blind method [mh] OR clinical trial [pt] OR clinical trials [mh] OR "clinical trial" [tw] OR comparative study [pt] OR evaluation studies [MeSH Terms] OR evaluation studies [mh] OR follow-up studies [mh] OR prospective studies [mh] OR cross-over studies [mh] OR treatment outcome [mh] OR "time series" [tw]

4) sensitive search

effect$ [tw] OR control$ [tw] OR evaluat$ [tw] OR compare$ [tw] program$ [tw] outcome$ [tw]

C) Identification of studies with relevant outcomes/or studies that include a work related term

5) work related outcomes:

"return to work" [tw] OR absenteeism OR "sickness absence" [tw] OR sick leave OR retirement OR "disability pension" OR "work disability" [tw] OR unemployment OR employment OR "work status" [tw] OR "work ability" [tw] OR "employment status" [tw]

6) OR "quality of life" [MeSH Terms] OR "quality of life" [tw]

7) OR fatigue [tw]

8) work related terms:

OR occupation$ OR "occupational health services" OR "occupational health" OR unemployed OR employed OR job OR vocational OR (occupational AND therap$) OR (occupational AND intervention$) OR "supported employment" OR "vocational rehabilitation" [tw] OR "work capacity evaluation" [tw] OR "vocational guidance" [tw] OR "work load"

## Pre-publication history

The pre-publication history for this paper can be accessed here:

http://www.biomedcentral.com/1471-2407/9/117/prepub
